# Newer-Generation Antidepressants and Suicide Risk

**DOI:** 10.1159/000502295

**Published:** 2019-09-05

**Authors:** Joseph F. Hayes, Gemma Lewis, Glyn Lewis

**Affiliations:** Division of Psychiatry, University College London, London, United Kingdom

We were interested in the letter by Hengartner and Plöderl [1] which was covered by the press in the United Kingdom. The issue of potential suicide risk in relation to antidepressant treatment is very important and we agree that the evidence for this is somewhat mixed [2, 3] and limited by low statistical power. Current guidelines recommend that clinicians should closely monitor patients for suicidal behaviour on initiation of treatment [4], particularly those under the age of 25 [2]. We agree with Hengartner and Plöderl [1] that the analysis by Khan et al. [5] is probably flawed because of the longer follow-up time in the antidepressant-exposed group, and the time-varying hazards of suicide and suicide attempts. There is some evidence that the possible increased risk for suicidality is still present after 2 weeks of SSRI use, but the main hypothesis suggests that the earlier effects of antidepressants are those that might lead to suicidal behaviour [6].

However, the analysis conducted by Hengartner and Plöderl [1] pooled data across all studies and this can potentially introduce bias and produce a misleading result [7]. A more statistically robust approach to aggregating data from different studies is to conduct a meta-analysis which produces an average of the results of each trial rather than simply pooling the results. We carried out meta-analyses of the data presented in table 1 of Hengartner and Plöderl's [1] letter using the *admetan* and *bayesmh* commands in Stata 15. We took a number of different analytical approaches: the DerSimonian and Laird model with 0.5 continuity correction, the inverse variance heterogeneity model with 0.5 continuity correction (both methods potentially lead to excess bias in the estimated effects and spuriously narrow CIs when outcomes are rare [8]), the Peto one-step OR model (the method recommended by Cochrane for rare events [9]), the Mantel-Haenszel model without zero-cell correction (which should perform better than the Peto method when treatment groups are unbalanced [8]), the reciprocal of opposite treatment arm correction (including both-armed zero event studies) and a Bayesian random effects model using an adaptive Metropolis-Hastings algorithm (using low-information prior distributions, so that data from the included trials dominated the final inferences; we used normal [mean = 0, variance = 100] for all means and gamma [0.0001, 0.0001] prior distributions for all precisions). We also include a simple pooled 2 × 2 table approach in Table 1, but as with Hengartner and Plöderl's [1] method, we believe this may have introduced bias given the larger ORs [7].

Our results are presented in Table 1. All the meta-analytic methods provide estimates well below Hengartner and Plöderl's [1] OR of 2.83 and we could not find any statistical evidence for an increased risk of suicide with antidepressants. Similarly, the number needed to harm calculated via this approach will include the possibility of no effect [10]. However, it is also important to point out that the statistical power and precision of all of these analyses is low. The confidence limits of all our calculations include potentially important risks of suicide associated with antidepressants. These data cannot rule out the possible increased risk of suicide with antidepressants, though we think there is much more uncertainty in the effect of antidepressants on suicide than originally suggested in Hengartner and Plöderl's [1] letter.

The main conclusion of Khan et al. [5] is that within placebo-controlled antidepressant trials, rates of suicide and suicide attempt have reduced in recent years, which is true whether analysed as patient exposure years or absolute numbers of events. This is most likely due to recruitment of individuals with lower baseline risk. This makes any extrapolation to the general population receiving antidepressants very challenging. For rare but serious adverse outcomes, minor methodological changes can impact on the certainty of the results and study populations are potentially very different from target populations. In those circumstances of uncertainty, clinical researchers should be cautious in communicating their results to the public.

## Disclosure Statement

Joseph F. Hayes and Gemma Lewis have no conflicts of interest to declare. Glyn Lewis has acted as an expert witness for litigation concerning antidepressants.

## Funding Sources

J.F.H. is funded by the Wellcome Trust (211085/Z/18/Z). All authors are supported by the NIHR UCLH Biomedical Research Centre. These funders played no role in the preparation of the manuscript.

## Author Contributions

Joseph F. Hayes and Gemma Lewis wrote the first draft of this correspondence. All authors edited the correspondence and agreed to submit it.

## Figures and Tables

**Table 1:** Different meta-analytic approaches to analysing suicide event data

Method	Summary OR (95% CI)
Mantel-Haenszel (without continuity correction)	0.72 (0.24–2.17)
Mantel-Haenszel (with continuity correction)	1.37 (0.60–3.15)
Inverse variance heterogeneity model (with continuity correction)	1.09 (0.46–2.58)
DerSimonian and Laird (with continuity correction)	1.09 (0.46–2.58)
Peto one-step	1.74 (0.78–3.90)
Reciprocal of opposite treatment arm correction (including both-armed zero event studies)	1.59 (0.72–3.53)
Bayesian meta-analysis	1.24 (0.50–2.32)^a^
Simple 2 × 2 table approach	2.93 (1.05–8.24)
Hengartner and Ploderl approach	2.83 (1.13–9.67)

a 95% credible interval.
